# Preparation of Phi29 DNA Polymerase Free of Amplifiable DNA Using Ethidium Monoazide, an Ultraviolet-Free Light-Emitting Diode Lamp and Trehalose

**DOI:** 10.1371/journal.pone.0082624

**Published:** 2014-02-05

**Authors:** Hirokazu Takahashi, Hiroyuki Yamazaki, Satoshi Akanuma, Hiroko Kanahara, Toshiyuki Saito, Tomoyuki Chimuro, Takayoshi Kobayashi, Toshio Ohtani, Kimiko Yamamoto, Shigeru Sugiyama, Toshiro Kobori

**Affiliations:** 1 Nanobiotechnology Laboratory, Food Engineering Division, National Food Research Institute, National Agriculture and Food Research Organization, Tsukuba, Ibaraki, Japan; 2 Isehara Research Laboratory, Technology & Development Division, Kanto Chemical Co., Inc., Isehara, Kanagawa, Japan; 3 Transcriptome Profiling Group, National Institute of Radiological Sciences, Chiba, Chiba, Japan; 4 Bio-Chemical Department, Reagent Division, Kanto Chemical Co., Inc. Tokyo, Japan; 5 Insect Genome Laboratory, National Institute of Agrobiological Sciences, Tsukuba, Ibaraki, Japan; Centro de Biología Molecular Severo Ochoa (CSIC-UAM), Spain

## Abstract

We previously reported that multiply-primed rolling circle amplification (MRPCA) using modified random RNA primers can amplify tiny amounts of circular DNA without producing any byproducts. However, contaminating DNA in recombinant Phi29 DNA polymerase adversely affects the outcome of MPRCA, especially for negative controls such as non-template controls. The amplified DNA in negative control casts doubt on the result of DNA amplification. Since Phi29 DNA polymerase has high affinity for both single-strand and double-stranded DNA, some amount of host DNA will always remain in the recombinant polymerase. Here we describe a procedure for preparing Phi29 DNA polymerase which is essentially free of amplifiable DNA. This procedure is realized by a combination of host DNA removal using appropriate salt concentrations, inactivation of amplifiable DNA using ethidium monoazide, and irradiation with visible light from a light-emitting diode lamp. Any remaining DNA, which likely exists as oligonucleotides captured by the Phi29 DNA polymerase, is degraded by the 3′-5′ exonuclease activity of the polymerase itself in the presence of trehalose, used as an anti-aggregation reagent. Phi29 DNA polymerase purified by this procedure has little amplifiable DNA, resulting in reproducible amplification of at least ten copies of plasmid DNA without any byproducts and reducing reaction volume. This procedure could aid the amplification of tiny amounts DNA, thereby providing clear evidence of contamination from laboratory environments, tools and reagents.

## Introduction

Phi29 DNA polymerase has the smallest size among known DNA polymerases, and is categorized as the B-family DNA polymerase. Intrinsic properties with Phi29 DNA polymerase, which are not found in other commercial DNA polymerases, make this polymerase especially unique and valuable [1]–[3]. Namely, Phi29 DNA polymerase has the highest processivity of DNA synthesis among commercial DNA polymerases (>70,000 nucleotides during a single binding event) [4] with the fastest rate of DNA synthesis (50–200 bases/s) without requiring any accessory protein [4], [5]. In addition, Phi29 DNA polymerase has high 3′ to 5′ exonuclease activity, which is involved in its high replication fidelity during DNA synthesis [6]–[9]. This high fidelity afforded by Phi29 DNA polymerase guarantees a rate of DNA replication errors that is nearly lesser than that of other DNA polymerases by 10^2^–10^3^ fold [8]–[10], resulting in a low rate of sequence mutation during DNA amplification [11]. Furthermore, Phi29 DNA polymerase has helicase-like activity coupled with DNA synthesis, called strand displacement activity [4]. Phi29 DNA polymerase continues to advance DNA synthesis while displacing downstream non-template DNA from a template. This strand displacement activity allows isothermal DNA amplification such as rolling circle amplification (RCA) [12], [13].

For more than a decade, there has been intense research into designing isothermal amplification techniques which can couple strand displacement using Phi29 DNA polymerase and exonuclease-resistant random primers. Two principal amplification methods have been developed: amplification of circular DNA such as plasmids, termed multiply-primed RCA (MPRCA) [14], and amplification of large linear DNA such as the human genome, termed multiple displacement amplification (MDA) [15]. In both methods, random hexamers significantly contribute to amplification of target DNA with unknown sequence, as is applied to amplification of genome DNA of a novel virus [16]–[19]. MPRCA and MDA also provide the highest amplification efficiency among various whole genome amplification (WGA) methods [11], [14], [15], [20]–[22]. Specifically, WGA by MDA is a game-changing technology for microbial genomics because MDA can accurately amplify DNA from tiny amount of a sample without sequence mutation. In fact, genome sequences are revealed using genome DNA amplified from non-culturable microbes living symbiotically in the gut of termites [23], [24]. Moreover, MDA has been applied to amplification of DNA isolated from a single microbial cell for further molecular biological analyses [25]–[32].

On the other hand, it is known that MPRCA and MDA are easily affected by exogenous contamination of DNA, as is often observed in the polymerase chain reaction (PCR) [33]–[53]. In particular, any DNA amplification in non-template control (NTC) does not ensure proper MDA reaction in test samples taking place in parallel 54,55. In reaction conditions free of exogenous DNA contamination, at least two additional factors affect the accuracy of amplification reactions: the self-annealing of random DNA primers [21], [56], [57], and endogenous DNA contamination of the reaction mixture [25], [58]–[60].

In some cases, byproducts derived from self-annealed random DNA primers are found in NTC despite a lack of DNA template in the reaction mixture [56], [57]. We have shown that modified random RNA primers, instead of the DNA primers, can effectively suppress non-specific DNA amplification in NTC [22]. This is because Phi29 DNA polymerase is a DNA-dependent DNA polymerase, which can use RNA as a primer for DNA synthesis but not as a template [22], [61]–[64].

DNA contaminants in reagents also affect amplification. The main source is DNA endogenously contaminating Phi29 DNA polymerase. Phi29 DNA polymerase has high affinity for both single stranded DNA (ssDNA) and double stranded DNA (dsDNA) [4]. Therefore, this endogenously contaminating DNA is presumably derived from the expression vector used for recombinant production of the polymerase and/or genome DNA of the host cells. Indeed, the *E. coli* genomic sequence is found in MDA products [25], [58]–[60]. To our knowledge, the level of DNA contamination in commercial Phi29 DNA polymerase differs from lot to lot, suggesting that each lot should be routinely checked prior to DNA amplification with Phi29 DNA polymerase.

Similarly, host DNA contamination has been reported in many PCR-based experiments, in particular the detection of bacterial DNA such as 16S rRNA genes. Various strategies aimed at eliminating contaminating DNA in PCR reagents have been reported and discussed [33]–[53], including DNase I treatment and short-wavelength ultraviolet (UV-C) irradiation; the latter strategy has also been used to prepare amplifiable DNA-free Phi29 DNA polymerase [25], [59], [60]. However, the polymerase itself may protect the contaminating DNA, perhaps captured by the polymerase, against DNase I in procedures such as DNase footprinting [47], [65]. In addition, UV-C treatment alone cannot degrade amplifiable contaminating DNA and instead may inhibit polymerase activity [43], [44], [46], [48]. It has been demonstrated that these procedures are ineffective in PCR [46], [50], [52], [53]. Therefore, in order to apply DNA amplification using Phi29 DNA polymerase to WGA and single cell genomics, a novel procedure for eliminating endogenously contaminating DNA in Phi29 DNA polymerase is required.

Here we describe a procedure for the preparation of Phi29 DNA polymerase containing significantly reduced amplifiable DNA. Crude Phi29 DNA polymerase was isolated by separation from host DNA, and followed by degradation of the remaining DNA by visible light (VL) irradiation in the presence of ethidium monoazide (EMA).

## Materials and Methods

### Solutions, Mixtures, Plasticware, Enzymes, DNA and Ribonucleotide

To avoid contamination by the laboratory environment, solutions supplied by manufacturers were used whenever possible. *Ultra*PURE™ distilled water (dDW) and glycerol were purchased from Invitrogen; 0.5 M ethylenediaminetetraacetic acid (EDTA) was from Nippon Gene; Tween 20 (molecular biology grade) and trehalose were from Kanto Chemical; Nonidet P40 (NP40, molecular biology grade) and polyethylenimine (PEI, 30% solution) were from Nacalai Tesque; 40% solution of tris(3-hydroxypropyl)phosphine (THP) was from Nippon Chemical Industrial, and 0.5 M THP solution was from Merck. All buffer solutions were made using dedicated pipettes in a dedicated laminar-flow cabinet (for enzyme preparation) after cleaning with RNase *AWAY*® (Molecular BioProducts). Most stock solutions, including the washing buffers, were filtered through a 0.1 µm filter unit (Nalgene).

Similarly, disposable, sterile plasticware (individually packed) was used whenever possible to reduce the chance of DNA contamination. Aerosol resistant filter tips were purchased from Molecular BioProducts; disposable plastic pipettes (2 ml, 5 ml, 10 ml and 25 ml, individually packed) were from Thermo Fisher Scientific; Eppendorf BioPur® microcentrifuge tubes (1.5 and 2.0 ml, individually packed) and PCR grade 0.2 ml tubes were from Eppendorf; conical tubes (15 ml and 50 ml) were from BD Falcon.

All restriction enzymes were purchased from Takara Bio; AccuRuler 1-kb DNA RTU ladder was from Maestrogen; Precision Plus Protein prestained standard (dual color) was purchased from Bio-Rad Laboratories; deoxynucleoside triphosphate (dNTPs) was from GE Healthcare. Thiophosphated hexaribonucleotide (6R5S; 5′-rN_S_rN_S_rN_S_rN_S_rN_S_rN-3′, HPLC grade) was purchased from Tsukuba Oligo Service. All oligonucleotide solutions and reaction mixtures for DNA amplification were prepared using a dedicated set of pipettes in a super-clean area (Class-1, according to International Standard Organization (ISO) 14644-1, Cleanrooms and associated controlled environments, Part 1: Classification of air cleanliness) produced using Table KOACH (Koken Ltd).

### Cloning of Phi29 DNA Polymerase into Expression Vector

The Phi29 DNA polymerase gene was amplified from a stock of bacteriophage ø29 [66], [67] by PCR using KOD plus DNA polymerase (Toyobo). PCR primers were designed based on GenBank accession number EU771092 such that *Eco*RI and *Not*I restriction sites were introduced at the 5′- and 3′- ends of the ORF. The sequences of the primers were as follows: 5′-gag aga gaa ttc *ATG* AAG CAT ATG CCG AGA AAG ATG TAT AG-3′; 5′-gag aga gag cgg ccg c *TT A*TT TGA TTG TGA ATG TGT CAT CAA CC-3′ (restriction sites underlined, ø29 gene in upper case, and start/stop codons in italics; Invitrogen). PCR was carried out in a total volume of 50 µL with 1.25 pmol of forward and reverse primer, 0.2 mM dNTPs, 1× KOD plus PCR buffer, 1 unit of KOD plus DNA polymerase. The PCR conditions were 96°C for 4 minutes followed by 30 cycles of 98°C for 20 seconds, 60°C for 10 seconds, and 72°C for 1.5 minutes, and completed with a final extension round for 4 minutes at 72°C using a TGradient thermocycler (Biometra). The PCR product was purified using a Wizard® SV Gel and PCR Clean-Up System (Promega). The resulting PCR fragment was digested and ligated into *Eco*RI-*Not*I cleaved expression vector pGEX-6P-1 (GE Healthcare) to yield the recombinant plasmid pGEX-*ø29pol* (approximately 6.7 kb). After transformation of *E. coli* strain DH5α (Toyobo), the plasmid DNA was purified from selected colonies and sequenced to check for mutations.

### Expression of GST fused Phi29 DNA Polymerase

*E. coli* strain BL21 (Novagen) was transformed with pGEX-*ø29pol* and grown on an LB agar plate (1% tryptone, 0.5% yeast extract, 0.5% NaCl, and 1.5% agar) containing ampicillin (50 µg/ml) to select the transformants. Cells were grown in LB medium (3 ml) at 37°C with shaking for 8 hours. A part of the culture was diluted 1∶1000 into fresh LB medium (250 ml) containing Overnight Express™ Autoinduction System (Novagen) and incubated at 30°C with shaking for over 16 hours. Cells were harvested in a 50 ml conical tube by centrifugation at 2,150×g for 15 minutes at 4°C and stored at −80°C until required.

### Decontamination and Isolation of Phi29 DNA Polymerase

Just before purification, 1 g of the cell paste was thawed at 4°C for over 30 minutes. The cells paste was resuspended in 5 ml of BugBuster® Protein Extraction Reagent (Novagen) containing 100 µL of 5 M NaCl, 100 units of Benzonase® nuclease (Novagen) and 2.0 mg of lysozyme (Nacalai Tesque) for 5 minutes in ice water. After the addition of an equal volume of DNA precipitation buffer containing 100 mM Tris-HCl (pH 7.5), 2 M NaCl, 20 mM EDTA, 4 mM THP, 1% Tween 20, 1% NP40 and 0.6% PEI, cell debris and host DNA were pelleted by centrifugation at 10,000 rpm for 15 minutes at 4°C. All subsequent steps were performed in a dedicated laminar-flow cabinet for protein purification. The supernatant was diluted with an equal volume of dilution buffer containing 66 mM Tris-HCl (pH 7.5), 1 mM THP, 10% glycerol, 1% Tween 20 and 1% NP40 to remove proteins bound to PEI. After centrifugation, 5 g of trehalose (approximately 0.66 M, Kanto Chemical) was dissolved into the clear supernatant.

The supernatant containing the recombinant GST-fused Phi29 DNA polymerase (GST-ø29pol) was then loaded onto glutathione Sepharose 4B resin (GS4B resin, GE Healthcare) using a Poly-Prep® Chromatography Column (GS4B column, bed volume 1 ml, Bio-Rad) as described in the manufacturer's protocol. The GS4B column was first washed with 25 ml of ice-cooled 2 M washing buffer containing 30 mM Tris-HCl (pH 7.5), 2 M NaCl, 1 mM EDTA, 4 mM THP, 1% Tween 20, 1% NP40 and 0.5 M trehalose to remove the other proteins. The GS4B column was washed with 250 ml of ice-cooled 3M washing buffer containing 10 mM Tris-HCl (pH 7.5), 3 M NaCl, 10 mM MgCl_2_, 1 mM EDTA, 4 mM THP, 1% Tween 20 and 1% NP40 to remove the remaining PEI. Then, the GS4B resin was resuspended in 2 ml of ice-cooled EMA treatment buffer containing 50 mM Tris-HCl (pH 7.5), 300 mM KCl, 1 mM EDTA, 4 mM THP, 1% Tween 20,1% NP40, and 10 µg/ml of EMA (Sigma-Aldrich), and the washed resin was transferred into a plastic petri dish (Ø 50 mm, As One). After gentle shaking for 30 minutes at 4°C in the dark, the GS4B resin in the petri dish was irradiated with VL using white-light emitting diode (LED) lamp (peak emission wavelength is approximately 455 nm, LDA9D-H, 60 W equivalent, Panasonic) positioned 5 cm above (304±23.2 mW/cm^2^, Light Meter LX-1108, Lutron) the dish for 60 minutes at 4°C with shaking. Then, the GS4B resin was transferred into a new Poly-Prep® Chromatography Column and washed with 250 ml of ice-cooled 0.3 M washing buffer containing 50 mM Tris-HCl (pH 7.5), 300 mM KCl, 1 mM EDTA, 4 mM THP, 1% Tween 20 and 1% NP40 to remove free EMA. After washing, the column was filled with phi29 buffer containing 50 mM Tris-acetate (pH 7.5), 25 mM magnesium acetate, 10 mM DTT, 0.5% Tween 20, 0.5% NP40, and 0.8 M trehalose and both ends of the column were capped. Then, the sealed GS4B column was incubated at 25°C for 3 hours to reduce the remaining oligonucleotides that might be bound to the Phi29 DNA polymerase. After incubation, the column was immediately washed with 25 ml of ice-cooled 0.3 M washing buffer at 4°C. The GS4B column was washed with 250 ml of ice-cooled 3 M washing buffer and 25 ml of 2× ice-cooled protease buffer containing 66 mM Tris-HCl (pH 7.5), 200 mM KCl, 0.2 mM EDTA, 1 mM THP, 1% Tween 20, and 1% NP40. Successive steps were performed in an ISO Class-1 environment produced using a Table KOACH (according to ISO 14644-1). Finally, the GS4B was resuspended in 1 ml of 2× ice-cooled protease buffer containing 40 units of PreScission™ Protease (GE Healthcare) and transferred into a BioPur® 2.0 ml tube (Eppendorf). After gently stirring for over 120 hours at 4°C, the Phi29 DNA polymerase fraction was collected (total 1.5 ml) from the GS4B resin using an empty mini column (Nacalai Tesque). Protein concentration was quantified using a DC™ Protein Assay kit (Bio-Rad) and spectrophotometry. After the addition of an equal volume of *Ultra*PURE™ glycerol and 0.002× volume of freshly prepared 1 M DTT, the purified Phi29 DNA polymerase fraction was transferred into new BioPur® 2.0 ml tube and stored at −20°C until use.

Cell lysate, supernatant after PEI precipitation, supernatant after dilution, GS4B resin (before and after digestion by PreScission™ Protease), purified protein (before and after addition of glycerol) and RepliPhi™ Phi29 DNA polymerase (Epicentre) were comparatively analyzed by SDS-PAGE using 10–20% gradient precast polyacrylamide gels (e-PAGEL, Atto Corporation) and visualized by staining with SimplyBlue™ SafeStain (Invitrogen). RepliPhi Phi29 DNA polymerase (Lot No. RPH-61004) was used as a control for the molecular weight and concentration of the native enzyme. The gel image was collected by a digital scanner (Epson, PM-A890) and analyzed using NIH Image software (ImageJ) to confirm the concentration of the purified Phi29 DNA polymerase.

### Preparation of DNase-treated Phi29 DNA Polymerase

DNase-treated Phi29 DNA polymerase was prepared essentially using the procedure described by Blainey and Quake [59]; however, our DNase-treated Phi29 DNA polymerase was obtained by separation from GST-tag.

### Verification of Contaminating DNA by RNA-primed MPRCA

To detect contaminating DNA in the polymerase, RNA-primed MPRCA was carried out with 10–10^4^ copies of pUC19 (Takara) or without the DNA template (*Ultra*PURE™ dDW). The RNA-primed MPRCA reaction was performed essentially as described previously [22]. Ten microliters of annealing buffer containing 20 µM 6R5S, 30 mM Tris-HCl (pH 7.5), 20 mM KCl, 8 mM MgCl_2_, and template DNA was denatured for 1 minutes at 95°C and cooled slowly to 25°C over 30 minutes. Then, 2× amplification premix (10 µL) was added, yielding a final concentration of 35 mM Tris-HCl (pH 7.5), 50 mM KCl, 14 mM MgCl_2_, 20 mM (NH_4_)_2_SO_4_, 5 mM DTT, 1 mM dNTPs, 0.002 U of inorganic pyrophosphatase (Roche) and 100 ng of the purified Phi29 polymerases or RepliPhi™ Phi29 DNA polymerase (Lot No. 10710). The reaction was performed for 16 hours at 30°C followed by incubation for 10 minutes at 65°C to inactivate the enzyme. To lower the viscosity of the amplification products, the products were transferred into 1.5 ml centrifuge tubes and mixed with 180 µL of dDW by pipetting, followed by vortex mixing. Twenty-five microliters of diluted products (12.5% of the original reaction product) were digested with 20 units of *Bam*HI and *Eco*RI (double-digested) for 1 hour at 37°C in a 50 µL volume. Then, 10 µL of each sample (2.5% of the original reaction product) was analyzed by electrophoresis using an agarose gel (1.0%, TAE buffer) and visualized by staining with ethidium bromide.

## Results

### Strategy for the preparation of Phi29 DNA Polymerase with considerably low amplifiable DNA

**Figure 1:**
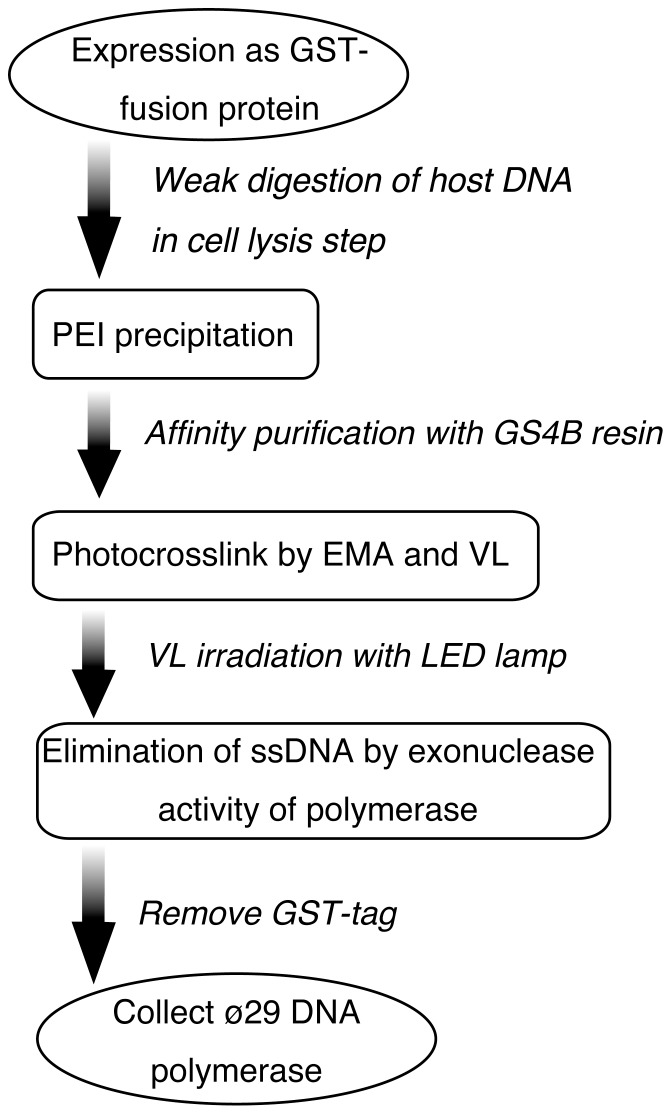
Schematic diagram of the DNA decontamination of Phi29 DNA polymerase. GST-fused Phi29 DNA polymerase (GST-ø29pol) is expressed in *E. coli* and affinity purified by glutathione sepharose 4B resin (GS4B) of the cell lysate after polyethylenimine (PEI) precipitation to remove the host DNA. Then, GST-ø29pol is treated with ethidium monoazide (EMA) and irradiated with visible light (VL) to reduce both ssDNA and dsDNA, which may be captured in GST-ø29pol. After washing away the free EMA, the remaining oligonucleotides are reduced by the endogenous 3′-5′ exonuclease activity of the polymerase. Finally, DNA-free Phi29 DNA polymerase is separated from GS4B by digestion with PreScission protease and collected.

Since ssDNA is a cause of primer-independent amplification, which is frequently observed in RCA using padlock probes [64], [68], we designed an effective procedure for decontaminating both ssDNA and dsDNA from Phi29 DNA polymerase. As illustrated in Figure 1, this strategy consists of three main steps: removal of host DNA; inactivation of DNA by photo-crosslinking; exonuclease treatment.

First, host DNA is rapidly separated from a homogenate of *E. coli* cells expressing Phi29 DNA polymerase as a GST fusion protein (GST-ø29pol). This Phi29 DNA polymerase has strong affinity for ssDNA but not dsDNA in the presence of over 100 mM NaCl [4], so GST-ø29pol binds to the free ssDNA or ssDNA region of dsDNA under these salt conditions. This suggests that minimizing the degradation of genomic DNA would decrease DNA contamination due to decreased binding of GST-ø29pol with ssDNA. Therefore, the host DNA is briefly digested with nuclease in salt conditions chosen so that the DNA has a viscosity slightly lower than that of the cell lysate. The digested DNA is immediately removed by PEI precipitation [69], and GST-ø29pol, which does not bind to DNA, is recovered in a supernatant fraction.

Second, a photo-crosslinking step comprising EMA and VL irradiation is used to inactivate the remaining dsDNA in GST-ø29pol, since DNA removal by PEI precipitation is incomplete. EMA (excitation wavelength, 456 nm) is intercalated into dsDNA, and subsequent VL irradiation leads to crosslinking of dsDNA [70]–[72]. This dsDNA crosslinked with EMA cannot be used as a template for DNA amplification [49], [50], [53], [70]–[72]. Since the molecular size of EMA is much smaller than that of any DNase molecule, EMA is more effective than DNase in inactivating dsDNA captured by the polymerase due to its easy access to the active site of GST-ø29pol. Furthermore, free EMA is easily removed from GST-ø29pol using an affinity column.

The third and final step is an exonuclease treatment. EMA treatment does not completely inactivate ssDNA, including oligonucleotides, as observed in PCR primers of PCR master mixes [49], [50], [53]. Therefore, remaining oligonucleotides are digested by the exonuclease activity of Phi29 DNA polymerase [6]–[9].

After these three steps, Phi29 DNA polymerase is removed from the affinity column by cleaving the GST-tag using a restriction protease. The purified Phi29 DNA polymerase, containing little amplifiable DNA, is then collected.

### Cloning, Expression and Purification of Phi29 DNA Polymerase

**Figure 2:**
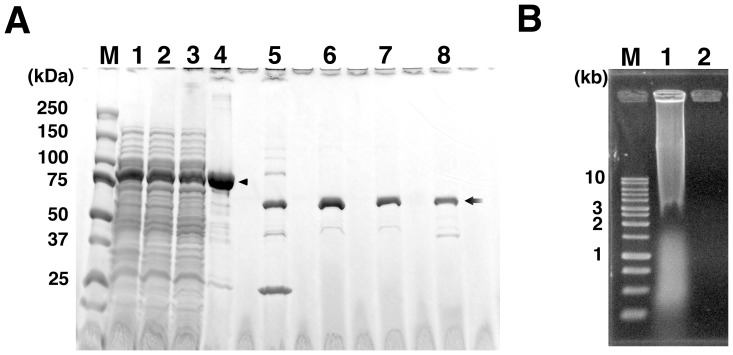
Isolation and purification of Phi29 DNA polymerase. (**A**) SDS-PAGE analysis of expressed and isolated GST-ø29pol, and purified Phi29 DNA polymerase. Lane; M, molecular mass markers (Bio-Rad); 1, whole cell lysate; 2, supernatant after PEI precipitation; 3, supernatant after dilution; 4, GS4B resin before digestion by PreScission; 5, GS4B resin after digestion by PreScission; 6, purified Phi29 DNA polymerase (before addition of glycerol); 7, purified Phi29 DNA polymerase (after addition of glycerol); 8, commercially available Phi29 DNA polymerase (1 µg, Epicentre, Lot No. RPH-61004). The arrowhead indicates the GST-ø29pol (calculated size, approximately 94 kDa) and the arrow indicates the purified Phi29 DNA polymerase and commercially available polymerase (calculated size, approximately 67 kDa). (**B**) Agarose gel analysis of the removal of host DNA by PEI precipitation. Lane; M, AccuRuler 1-kb DNA ladder (Maestrogen); 1, whole cell lysate; 2, supernatant after PEI precipitation.

*E. coli* BL21 strain was transformed with an expression vector, pGEX-*ø29pol*, harboring a gene encoding Phi29 DNA polymerase, and expression of GST-ø29pol (approximately 94-kDa) was verified by SDS-PAGE analysis of the whole cell lysate (**Fig. 2A** lane 1). Following this, approximately 2 g of the overexpressed cells were prepared from large-scale culture using autoinduction systems for further purification.

The collected cells was lysed in BugBuster with benzonase in a buffer containing 100 mM NaCl to gradually degrade the host genomic DNA. Benzonase remained active under this salt condition, and successfully lowered the viscosity of the lysate. Host DNA and cell debris were immediately precipitated with PEI and high salt condition (1 M NaCl). Benzonase got inactive under the high salt condition, so the host DNA was not further fragmented and was easily precipitated with PEI [69]. The resulting soluble GST-ø29pol was recovered in the supernatant with little contaminating host DNA (**Fig. 2A** lane 1 and 2 and **Fig. 2B** lane 1 and 2). This supernatant was used for affinity column purification using GS4B resin.

However, soluble GST-ø29pol easily aggregated (data not shown), even when the purification step was performed at 4°C. The aggregated proteins clogged the column and retarded flow, resulting in low yield of purified Phi29 DNA polymerase. Non-ionic detergents to prevent aggregation did not work. Other reagents that prevent or disrupt aggregation, such as arginine [73], [74], the non-detergent sulfobetaine (NDSB) series [75], [76] and trehalose [77], [78], were also tested. The aggregation of GST-ø29pol was suppressed by 0.66 M trehalose or higher concentrations, allowing the isolation of GST-ø29pol using a GS4B affinity column. As a result, GST-ø29pol was collected as the major product on the GS4B resin (**Fig. 2A** lane 4, arrowhead, approximately 94 kD).

Subsequently, GST-ø29pol bound to the GS4B resin was then treated with EMA, followed by exposure to a white-LED lamp to inactivate the remaining dsDNA. DNA amplification experiment shows that DNA remaining in the sample or binding to the polymerase was not effectively inactivated by the exposure for less than 30 min (**Figure S1**). This is presumably because that easy precipitation of GS4B resin would inhibit homogeneous exposure to the white light for less than 30 min even if the sample kept being suspended by rotator. Based on this result, irradiation time was designed to be 60 min in our procedure. After washing out the free EMA, the remaining DNA was digested using the 3′-5′ exonuclease activity of GST-ø29pol in 0.8 M trehalose. These buffer conditions retain the activities of GST-ø29pol, including its 3′-5′ exonuclease activity [79].

Finally, Phi29 DNA polymerase was recovered from GS4B using restriction protease (**Fig. 2A** lane 5, 6 and 7). The purified polymerase was approximately 67 kDa, which was the same as that of a commercial Phi29 DNA polymerase (**Fig. 2A** lane 8). The average yield of the purified Phi29 DNA polymerase was approximately 1.5 mg per gram of pelleted cells.

### Verification of the amplifiable DNA-free Phi29 DNA polymerase

**Figure 3:**
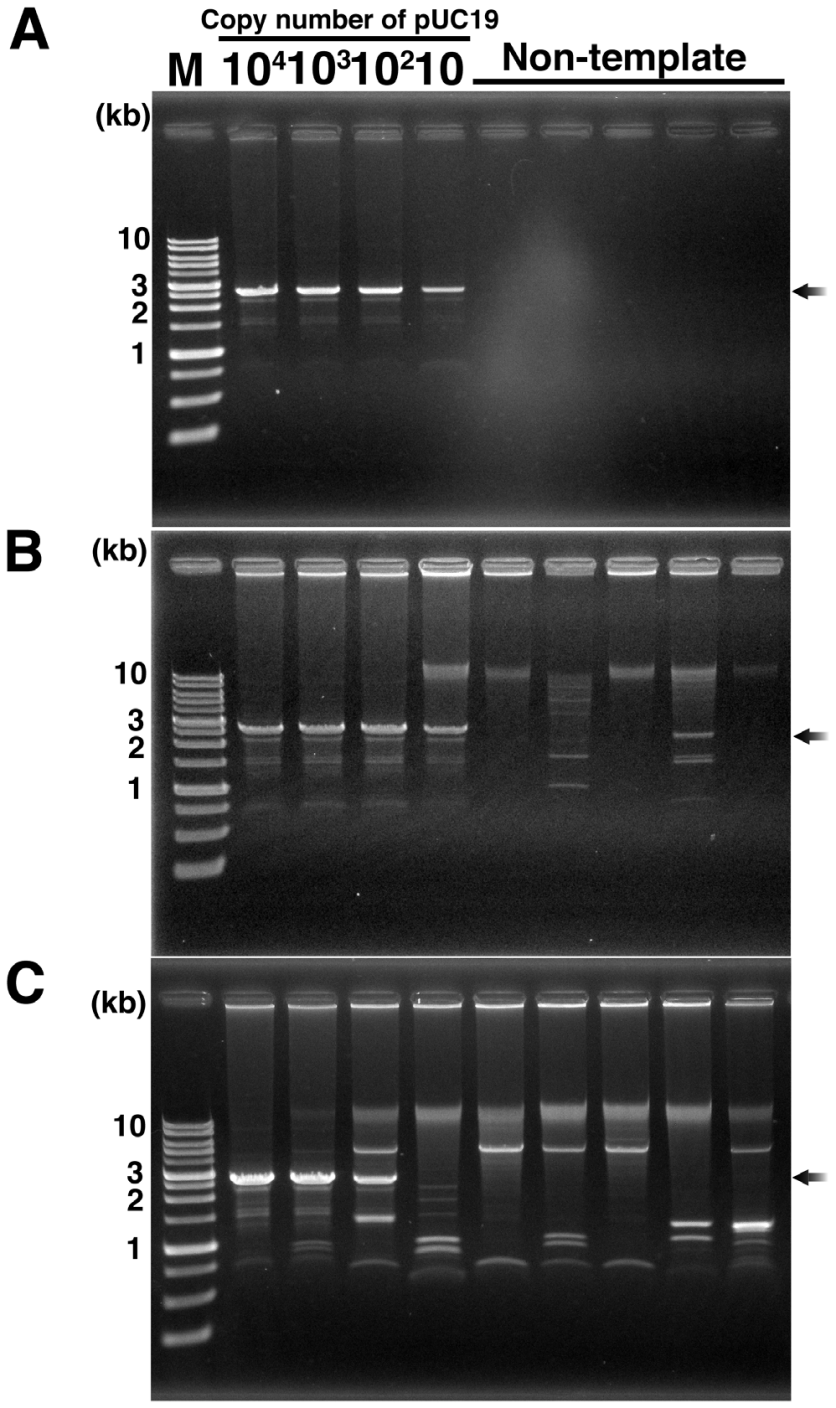
Gel analysis of RNA-primed MPRCA products to detect the contaminating DNA in Phi29 DNA polymerase. (**A**) DNA amplification with Phi29 DNA polymerase prepared by our procedure. (**B**) DNA amplification with commercially available Phi29 DNA polymerase (Epicentre, Lot No. 10710). (**C**) DNA amplification with Phi29 DNA polymerase prepared by DNase-treatment. All gel images show the *Bam*HI/*Eco*RI double digested amplification products. Black arrow indicates the specific amplification products from pUC19; M, AccuRuler 1-kb DNA RTU ladder (Maestrogen). All reactions were repeated three times or more; typical results are shown.

We verified that the purified Phi29 DNA polymerase contained little amplifiable DNA, including plasmid and genomic DNA. Current MPRCA using protected RNA primers reproducibly amplify at least ten copies of plasmid DNA without any byproducts in NTC [22]. In addition, since MPRCA and MDA use the same DNA amplification machinery, RNA-primed MPRCA can also amplify DNA fragments, (e.g., from the *E. coli* genome) contaminating in the polymerase. The amplification products were confirmed by agarose gel electrophoresis after *Bam*HI/*Eco*RI double-digestion because specifically amplified products from pUC19 would be found as an obvious band corresponding to the linear form of pUC19 (approximately 2.7 kb) by the digestion [21], [22]. However, non-specific amplification products emerge as fragments with other sizes, or are migrated at over 20 kbp [21], [22]. As a result, no amplification product were found in plural NTCs using the purified Phi29 DNA polymerase, while 2.7 kb fragment was found in all positive controls using pUC19 as a template DNA (**Fig. 3A**). This result indicates that this purified Phi29 DNA polymerase contains little amplifiable DNA, and that RNA-primed MPRCA using this purified Phi29 DNA polymerase has the potential to reproducibly amplify ten copies of pUC19 (approximately 30 attograms, **Fig. 3A**) without any byproducts and reducing reaction volume as previously reported [22].

By contrast, non-specific amplification products were found in all NTCs and positive controls using the commercially available Phi29 DNA polymerases (Epicentre Lot.No. 10710, **Fig. 3B**) and DNase-treated Phi29 DNA polymerase prepared as described by Blainey and Quake [59] (**Fig. 3C**). This result suggests that contaminating DNA in Phi29 DNA polymerase is protected by the polymerase from DNase degradation, as observed in DNase footprinting experiments [47], [65]. Furthermore, these results also suggest that DNA might be additionally contaminated by DNase-treatment, as observed in previous reports on PCR-decontamination procedures [33], [46], [47]. Moreover, the limit of specific amplification using our DNase-treated polymerase was reduced to 10^3^ copies of pUC19 (approximately 3 femtograms, **Fig. 3C**). This result also indicates that contaminating DNA in the polymerase reduces the efficiency of the amplification of the target DNA. The evidence presented above indicates that Phi29 DNA polymerase purified by our procedure contains less amplifiable DNA and demonstrates higher amplification efficiency than the other two polymerases tested.

## Discussion

DNA amplification with Phi29 DNA polymerases, such as MPRCA and MDA, holds promise for analyzing unknown biological functions and discovering novel substances through WGA and single cell genomics without any cultivation step [80], [81]. Based on the predicated molecular mechanism for MPRCA and MDA, short linear DNA cannot be amplified by these methods [56], [82], [83], but this is clearly contradicted by the observation that template-independent byproducts such as oligonucleotide primers, including DNA hexamers, are observed [22], [56], [57], [64], [68]. It remains unclear how byproducts are produced from short linear DNA. Regardless, such template-independent amplification remains a serious problem which must be solved in order to make WGA and single cell genomics feasible and practical [25]–[27], [58]–[60], [80], [81], [84]–[86]. Here, we have demonstrated a significant decrease in the levels of dsDNA and ssDNA endogenously contaminating Phi29 DNA polymerase.

Host DNA is a major source of DNA contamination during the preparation of recombinant polymerase [52]. Since DNA polymerases generally have high affinity for both dsDNA and ssDNA [47], the host DNA must be separated from the produced polymerase immediately following cell lysis step. Subsequently, benzonase digestion of the host DNA is necessary to lower the viscosity of the sample, but this alone is insufficient for complete digestion of the host DNA to mononucleotides, which would not affect byproduct amplification in NTC. Over-digestion of host DNA increases the amount of DNA captured by Phi29 DNA polymerase. Therefore, our procedure controlled the level of DNA digestion so as to maximize the efficiency of host DNA removal by successive PEI precipitations. Since Phi29 DNA polymerase preferentially binds to ssDNA rather than dsDNA [4], it was impossible to completely eliminate the contaminating DNA by PEI precipitation, in particular DNA bound to the Phi29 DNA polymerase active site. Indeed, Phi29 DNA polymerase treated with a combination of benzonase and PEI still gave byproducts in NTC of MRPCA, leading us to speculate that DNA captured by Phi29 DNA polymerase was the cause of template-independent amplification.

EMA and VL irradiation were employed to inactivate the captured DNA after affinity chromatography. EMA is known to intercalate with dsDNA, and successive VL irradiation leads to photo-crosslinking of dsDNA. Indeed, EMA-treatment is an efficient procedure for eliminating contaminating DNA in PCR reagents [49], [50], [53]. In addition, it is reported that photo-activated free EMA can also degrade ssDNA [87]; therefore, we expected that EMA treatment could reduce both dsDNA and ssDNA contaminants in the Phi29 DNA polymerase fraction. However, as observed in PCR with EMA-treated reagents [49], [50], [53], EMA treatment for GST-ø29pol did not completely inactivate ssDNA in our decontamination procedure. Although photoactiovated EMA may digest ssDNA to many pieces of oligonucleotides, it cannot completely degrade ssDNA down to mononucleotides that do not affect amplification results of WGA using Phi29 DNA polymerase. Accordingly, any remaining ssDNA, including oligonucleotides, were digested by the endogenous exonuclease activity of the polymerase.

Propidium monoazide (PMA) is also used for decontamination of PCR reagent [49], suggesting that PMA may be a substitute for EMA in our procedure. A comparison of molecular weight of EMA with that of PMA, however, indicates that EMA would effectively contribute to inactivation of contaminating DNA captured in the binding site of Phi29 DNA polymerase than PMA due to easier access to the binding site.

In our procedure, light source is important because conventional lamps (e.g. halogen lamp) for VL irradiation emit light covering both VL and UV, and eventually heat the polymerase fraction. Our preliminary studies show that UV-C seriously damages polymerase activity and that the elevated temperature considerably induces polymerase aggregation. VL generated by LED lamp covers a limited range of wavelength and suppresses temperature elevation. Indeed, our procedure required these technical advantages of LED lamp for successful decontamination.

On the other hand, irradiation period of our procedure is much longer than that of conventional viability-PCR (vPCR) method using dedicated blue-LED lamp [72]. When we had tried DNA amplification using polymerases purified after short-irradiation times (10 and 30 min), instability for results of plural NTCs in WGA was clearly observed (**Figure S1**), while a polymerase purified via 60-min irradiation did not give any DNA amplification in all NTCs (**Fig. 3A**). These results suggest that 30-min irradiation would not be suitable for light-induced interaction of DNA with EMA in our procedure for decontamination of polymerase. Considered that the irradiating light is free of UV region and that the step is performed at 4°C, purified polymerase could maintain its activity as observed in Fig. 3A, even if the irradiation time is 60 min. Based on a series of experiments regarding dependence of irradiation time, therefore, our procedure was designed to include 60-min irradiation of white-LED.

Our study eventually clarified that irradiation time of vPCR would not suit decontamination procedure of phi29 DNA polymerase. In vPCR, template DNA isolated from dead cells should become shorter than amplicon size by photoactivation of EMA, and it is not necessary to be completely broken down to mononucleotides. On the other hand, a result of WGA is easily affected by shorter DNA that could be primers or templates for WGA using phi29 DNA polymerase and random primers, even though the DNA does not possess suitable size for vPCR template. Therefore, a difference in amplification mechanism between WGA and vPCR would need longer irradiation of white-LED in our procedure than vPCR.

Even in vPCR, irradiation time also depends on the sample condition (e.g, color, volume and turbidity), light source, irradiation procedure (e.g. distance from light source), material of plasticware, and amplicon size [72]. Commercial device is optimum for only vPCR, but not optimized for decontamination procedure of phi29 DNA polymerase that contained many GS4B resin (white color and high-turbidity). Intensity of light at 456 nm from white-LED lamp is much lower than that of the blue-LED lamp, and the light is easily diffused by the samples containing the resin which inhibits even irradiation due to its difficult suspension in dish by rotator. Lines of evidence, therefore, indicate that longer irradiation to EMA in the purification than in vPCR would be derived from differences in DNA amplification mechanism, sample condition, and intensity of light source.

While the purification conditions were being optimized, we realized that Phi29 DNA polymerase often aggregated, even when all the purification steps were performed at 4°C. Aggregated polymerase easily bound to GS4B resin and could not be eluted. In addition, the association of Phi29 DNA polymerase with other proteins during the affinity purification step seemed to pull benzonase into the final fraction, leading to template degradation in successive amplification reactions. A screening of anti-aggregation reagents shows that trehalose worked well and improved the final yield of the pure polymerase. In addition, trehalose facilitates digestion of the remaining oligonucleotide by the innate exonuclease activity of Phi29 DNA polymerase, while at the same time reduces the aggregation at 25°C condition. Thus, preventing aggregation using trehalose is also critical in our purification procedure.

In conclusion, the removal of contaminating DNA from Phi29 DNA polymerase was achieved by combining several readily available techniques including tag-fusion, EMA, LED irradiation and trehalose. This treatment significantly lowered amplifiable-DNA in the polymerase without loss of polymerase activity. This suggests that this versatile procedure could be easily applied in general to the DNA decontamination of both mesophilic and thermophilic polymerases [49], [50], [53]. Thus, this procedure could contribute significantly to the amplification of tiny amounts DNA and provide clear evidence of contamination from laboratory environments, tools and reagents.

## Supporting Information

Figure S1 Gel analysis of RNA-primed MPRCA products to detect the contaminating DNA in Phi29 DNA polymerase prepared with reducing VL irradiation times. All gel images show the *Bam*HI/*Eco*RI double digested amplification products. M, 1-kb DNA ladder (Sigma-Aldrich); P, positive control (pUC19, 10^6^ copies); NTCs, Non-template controls. Numbers in panels indicate lot numbers (preparation date) of Phi29 DNA polymerase.
(TIF)
