# Supplementary figures and images for: Preparation of Phi29 DNA Polymerase Free of Amplifiable DNA Using Ethidium Monoazide, an Ultraviolet-Free Light-Emitting Diode Lamp and Trehalose
Source: PLoS One. 2014 Feb 5;9(2):e82624. doi: 10.1371/journal.pone.0082624 (PMC3915000; doi:10.1371/journal.pone.0082624)

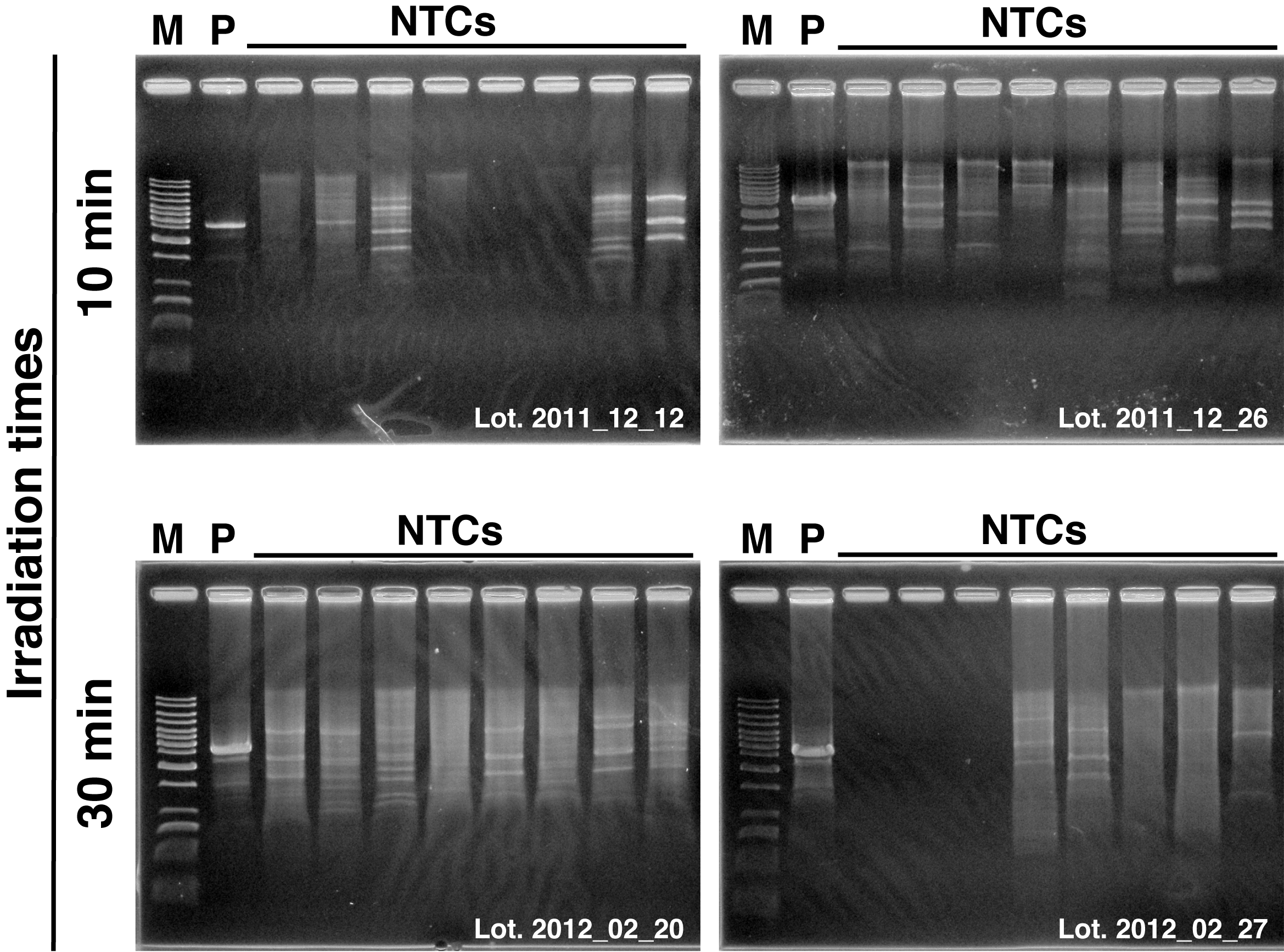

Supplement: Figure S1 — Gel analysis of RNA-primed MPRCA products to detect the contaminating DNA in Phi29 DNA polymerase prepared with reducing VL irradiation times. All gel images show the BamHI/EcoRI double digested amplification products. M, 1-kb DNA ladder (Sigma-Aldrich); P, positive control (pUC19, 106 copies); NTCs, Non-template controls. Numbers in panels indicate lot numbers (preparation date) of Phi29 DNA polymerase. (TIF) [file pone.0082624.s001.tif]
